# Non-invasive synchronous monitoring of neutrophil migration using whole body near-infrared fluorescence-based imaging

**DOI:** 10.1038/s41598-021-81097-8

**Published:** 2021-01-14

**Authors:** Jack Leslie, Stuart M. Robinson, Fiona Oakley, Saimir Luli

**Affiliations:** 1 Newcastle Fibrosis Research Group, Faculty of Medical Sciences, Biosciences Institute, Newcastle University, Newcastle upon Tyne, UK; 2 Department of Hepatobiliary Surgery, Newcastle Upon Tyne Hospitals NHS Foundation Trust, Newcastle upon Tyne, UK; 3 Faculty of Medical Sciences, Preclinical In Vivo Imaging, Biosciences Institute, Newcastle University, 4th Floor, William Leech Building, Framlington Place, Newcastle upon Tyne, NE2 4HH UK

**Keywords:** Imaging the immune system, Chemotaxis, Fluorescence imaging, Optical imaging

## Abstract

Advances in fluorescence imaging coupled with the generation of near infrared probes have significantly improved the capabilities of non-invasive, real-time imaging in whole animals. In this study we were able to overcome a limitation of in vivo fluorescence imaging and have established a dual cell tracking method where two different cell types can be monitored according to the spectral signature of the cell labelling fluorophore. Using a mouse model of acute liver injury, we have characterised the in vivo migration patterns of wild type and transgenic neutrophils with impaired chemotaxis. Here, we were able to demonstrate that IVIS provides a sensitive multiplexing technology to differentiate two different cell populations based on the spectral signature of the cell labelling fluorophores. This spectral unmixing methodology has the potential to uncover multidimensional cellular interactions involved in many diseases such as fibrosis and cancer. In vivo spectral un-mixing provides a useful tool for monitoring multiple biological process in real-time in the same animal.

## Introduction

Inflammation is a major factor in the development of many chronic diseases and cancer. Targeting immune cells is an emerging therapeutic approach for such disorders and recent advances in immune and stem cell-based therapies have increased the use of non-invasive longitudinal imaging of live cells in animal models^1^. Compared to traditional methods such as histology, in vivo imaging allows non-invasive, longitudinal qualitative and quantitative measure of a therapeutic or biological effect. In vivo imaging modalities offer temporal and spatial live cell monitoring, which provides a detailed insight into chemotaxis, homing and engraftment of cells to a particular organ as well as studying cell behaviour^2^.

In vivo imaging technologies rely directly on the development of labelling molecules in order for the cells to be visualised in real time in a whole animal. Positron-Emission Tomography (PET) and Single-Photon Emission Tomography (SPECT) use ionising radiation as a cell marker, whilst, Magnetic Resonance Imaging (MRI) exploits contrast agents that produce a magnetic spin to visualise cells in a small animal^3^. Unlike MRI and PET/SPECT, optical imaging detects photons emitted from fluorophores that have distinct spectral signatures^4^. Therefore, in vivo fluorescence imaging modalities, theoretically could facilitate synchronous multiplex imaging of various probes with different spectral properties. However, the resolution of such imaging systems is limited by tissue depth, light scatter and auto-fluorescence. Advances in chemical engineering of fluorescent probes to emit in the Near Infrared Region (NIR) of the light spectrum has dramatically minimised the effect of auto-fluorescence and tissue absorption whilst maximising photon penetrance and image resolution^5^. Signal to noise ratio (SNR) in optical imaging systems has further improved due to recent innovations in charge coupled device cameras and emission/excitation filters^6,7^. In addition, development of spectral un-mixing algorithms has provided a unique opportunity to further enhance contrast and sensitivity by separating pure signal from auto-fluorescence^8^. The simplicity, cost efficiency and the ability to image multiple animals at the same time make optical imaging ideal for minimally invasive longitudinal cell tracking experiments^9^.

Standard in vivo imaging modalities are typically capable of monitoring a single defined cell population. Alternative methods such as microscopy and flow cytometry, allow monitoring of multiple cell populations however, these methods only provide a snapshot picture of temporally regulated processes such as cell chemotaxis^10,11^. Using current in vivo imaging methods to compare the dynamics and the interplay between multiple cell populations requires that each cell type is injected into different recipient mice, which does not control for recipient inter-variability or the efficiency of cell administration^9^. Nevertheless, there are reports in the literature demonstrating in vivo fluorescent imaging of two (or more) different cell populations in the same animal. However, these studies often utilise novel proprietary fluorophores developed in advanced molecular photonics laboratories^12^ and/or have often been performed in nude or albino BALB/c mice, with the cells injected subcutaneously in distinct locations^13,14^ in order to minimise imaging depth and signal attenuation by dark skin pigmentation and black fur. However, C57BL/6 mice are often the preferred strain for preclinical mouse models because the majority of transgenic mice are generated on this background.

In order to develop a novel in vivo imaging methodology capable of discerning the spectral signature of two different NIR dyes in a deep-seated organ we utilised both wild type (WT) mice and mice deficient for the c-Rel subunit of transcription factor NF-κB (*Rel*^*−/−*^ mice), which have known defects in immune cell recruitment to the liver following injury^15^. Using WT C57BL/6 and *Rel*^*−/−*^ mice we have optimised a novel dual fluorescence imaging (DFI) technique but also highlighted an intrinsic defect within *Rel*^*−/−*^ neutrophils which is responsible for their failed chemotaxis.

## Materials and methods

### Mice

All animal experiments were approved by the Newcastle University Ethical Review Committee and performed under a UK Home Office licence in accordance with the ARRIVE guidelines (http://www.nc3rs.org.uk/page.asp?id=1357). Experiments were performed on either C57BL/6 wild-type (WT) control miceor c-Rel knockout mice (*Rel*^*−/−*^). *Rel*^*−/−*^ mice which lack the *Rel* gene were a kind gift of Hsiou Chi-Liou (Weill Cornell Medical College).

### Acute carbon tetrachloride liver injury

Administration of carbon tetrachloride (CCl_4_) is a commonly used hepatoxic agent for induction of acute and chronic liver injury. In an acute model, CCl_4_ is diluted with olive oil vehicle at ratio of 1:1 and given as a single intraperitoneal injection (CCl_4_:Olive Oil, 1:1 [v:v]) at a dose of 2 μl/g of body weight. At this concentration a single dose of acute CCl_4_ is sufficient to cause significant hepatocyte damage and promote increased expression of inflammatory cytokines and chemokines to recruit immune cells to the injured liver^16^. Control mice received a single intraperitoneal injection of olive oil vehicle at a dose of 1 μl/g body weight. Following in vivo imaging mice were humanely killed for ex vivo imaging and tissue processing.

### Histology

5 µm-thick formalin-fixed, paraffin-embedded (FFPE) liver sections were processed for haematoxylin and eosin (H&E). All stains were analysed at 10 × magnification using a Nikon Eclipse Upright microscope and NIS-Elements BR analysis software.

### In vivo fluorescence imaging

WT and *Rel*^*−/−*^ recipient mice received a single intraperitoneal dose of CCl_4_ 24 h prior to the intravenous injection of fluorescently labelled cells to be tracked. Direct cell membrane labelling of neutrophils was performed according to manufactures instructions using CellVue 710 nm (burgundy dye, Thermo Fisher Scientific, product code 88-0872-16), 780 nm labelling and CellVue NIR815 dyes (Thermo Fisher Scientific, product codes 88-0875 and 88-0874-16 respectively). The neutrophils were administered by an intravenous injection of 100 μl of cell suspension/mouse. Mice under isoflurane anaesthesia were fluorescently imaged using the following excitation and emission filters: 675/720 nm for the burgundy dye, 745/800 nm for 780 nm dye and 745/820 for NIR815 dye. Epi-fluorescent images were acquired on an IVIS Spectrum (Caliper Life Sciences) at 2 h post cell administration. Following in vivo imaging, mice were humanely killed, the livers were excised and then fluorescently imaged using the IVIS Spectrum (using above filters). For maximum signal sensitivity, all images were acquired under the same field of view using autoexposure, which corresponded to pixel binning of 8 and f/Stop of 2. Changes to the acquisition parameters that affects sensitivity, exposure time /f-stop/ binning and field of view is automatically normalized by the Living Image software.

### Image analysis

Data was analysed using Living Image version 4.5.2 and 4.7.2 software (https://www.perkinelmer.com/uk/lab-products-and-services/resources/in-vivo-imaging-software-downloads.html). For in vivo imaging, the liver region was defined as area between the sternum and middle of the abdominal area. Regions of interest (ROIs) were drawn in the defined area and quantified in the physical, calibrated unit "Radiant Efficiency [p/s/sr]/[µW/cm^2^]". The Living Image software normalises automatically for sensitivity differences resulting from different exposure times without any user input required, when ROI values are expressed in a calibrated, physical unit. For fluorescent imaging the calibrated unit is "Radiant Efficiency", for bioluminescent imaging "Radiance/photons".

Tissue autofluorescence (TAF) of the mice was measured by imaging mice with filter sets appropriate for each cell labelling dye prior to administration of fluorescently labelled cells. The ROI values of the tissue autofluorescence were subtracted from the ROI values measured after injection of the labelled cells (True signal = Raw Signal − TAF).

Because we report ROI values as "Average Radiant Efficiency" [p/s/cm^2^/sr]/[µW/cm^2^] (= Radiant Efficiency/ROI surface area) the ROI’s were kept the same size for each mouse (in vivo) or organ (ex vivo) within a study group and were appropriately sized to encompass the fluorescent signal for all mice, to ensure that the imaging data between individual donors can be compared within a study group.

Spectral unmixing was performed using Living Image software, which benefits from a proprietary algorithm specifically designed by the manufacture for the IVIS system. Within Living Image software, the spectral unmixing module was utilised to separate the two dyes. Living Image benefits from a multiplexing algorithm which is derived from a multivariate curve resolution (MCR) method^8^.

Identification of the optimal excitation and emission filters to achieve fluorophore separation between the Burandy and 780 nm dyes was achieved by initially imaging cells labelled with each dye and performing image acquisition across varies filter sets (excitation / emission). This allowed us to obtain the pure spectral signature of each dye. The spectral properties of both fluorophores were stored into a library within living image software. To differentiate cells based on cell labelling dye, the spectral properties of each fluorophore were applied to all other images. Composite images were generated using the image overlay function on Living Image.

### Bone marrow derived neutrophil isolation

Neutrophils were isolated from donor WT or *Rel*^*−/−*^ mice. Typically, 5 million cells were isolated from one donor mouse. Briefly**,** the femur and tibia from both hind limbs were harvested and then the bone marrow was flushed out. Red blood cells were then lysed, and the cell suspension was passed through a 100 μm filter. Neutrophils were isolated using a 62.5% Percoll density gradient in phosphate buffered saline (PBS). The mixture was centrifuged for 30 min at 1000 g with no brake or acceleration. Cells were then resuspended and counted. Prior to administration into recipient WT or *Rel*^*−/−*^ mice, cells were labelled with appropriate cell labelling dye.

### Fluorescent cell labelling

Following neutrophil isolation, cells were washed in serum free media. Cells were counted and resuspended at concentration of 2 × 10^7^ cells/mL in Diluent C (supplied in the CellVue labelling kit, Thermo Fisher Scientific). For a final labelling concentration of 2 μM of dye, a working solution was prepared by adding 4μL of the 1 mM dye stock to 1 mL of Diluent C. Cells and dye solution were mixed together followed by a 3-min incubation time at room temperature. Cell labelling was stopped by adding an equal volume of 100% fetal bovine serum (FBS) and incubated for 1 min at room temperature. Cells were then washed by centrifugation and the supernatant was discarded. The cell pellet was resuspended in complete media and then washed a further 3 times in PBS to remove residual Diluent C and any unbound dye prior to intravenous administration.

The above protocol was applied for both CellVue NIR815 (Thermo Fisher Scientific catalogue number 88-0874-16) and CellVue Burgundy (Thermo Fisher Scientific, catalogue number 88-0872-16). However, for the CellVue NIR780 (Thermo Fisher Scientific, catalogue number 88-0875-16) a final concentration of 4 μM was used to achieve the same IVIS signal to that of CellVue Burgundy.

### Isolation of immune cells from mouse hepatic tissue

Upon completion of in vivo and ex vivo imaging, leukocytes were isolated from the excised livers of recipient WT or *Rel*^*−/−*^ mice. Livers were finely diced and then digested for 1 h at 37 °C using 25 μg/ml DNase and 1 mg/ml Collagenase B. This was then passed through a 70 µm cell strainer and then layered on top of a 33% Percoll density gradient and centrifuged for 30 min at 1000*g* with no brake. The red blood cells were then lysed prior to labelling for flow cytometric analysis.

### Flow cytometry

Flow Cytometry was performed to determine the number of fluorescently labelled neutrophils accumulated in the injured liver to validate the IVIS signal at a cellular level. Single cell suspensions were first resuspended in LIVE/DEAD Fixable Violet Dead Cell Stain (ThermoFisher) and then Fc blocked (CD16/32). Cells were then resuspended in Flow buffer (PBS 1% FCS) containing the CD45 (BV510 Biolegend 103138), CD11b (PerCP-Cy5.5 Biolegend 101228) and Ly6G (APC Biolegend 560596) antibodies. Cells were read on a BD LSRFortessa X-20 (BD Biosciences) using FACSDIva v8 (https://www.bdbiosciences.com/us/instruments/clinical/software/flow-cytometry-acquisition/bd-facsdiva-software/facsdiva-software-v-803-win-7-32-bit-os/p/659523) and analysed using FlowJo v10 (https://www.flowjo.com/solutions/flowjo/downloads). 710 nm (CellVue Burgundy) positive cells were observed in the channel 640–730/45 nm, whereas the 780 nm (CellVue NIR780) positive cells were observed in 640–780/60 nm channel.

### Liver function test

Serum transaminases levels of alanine aminotransferase (ALT) and aspartate aminotransferase (AST) were measured at the clinical pathology department in the Royal Victoria Infirmary, Newcastle upon Tyne.

### Statistical analysis

Results are presented as mean ± s.e.m. Graphpad prism 8 (https://www.graphpad.com/scientific-software/prism/) was used to perform the statistical analyses. Statistically significant differences were calculated using either a two-tailed unpaired Student t test or a Two-way Anova with a Tukey’s post hoc test, *P < 0.05, **P < 0.01 or ***P < 0.001 was considered statistically significant.

## Results

### In vivo fluorescent imaging permits monitoring of immune cell recruitment in C57BL/6 mice

In vivo whole body fluorescent cell tracking in nude and albino mice is a well-established methodology and is routinely used to monitor cell migration in mouse models, typically where the injury is superficially located^17^. However, due to limitations of tissue depth, signal attenuation by skin pigmentation and black fur, in vivo fluorescence imaging in deep-seated organs of C57BL/6 mice is less routinely performed^18^. To demonstrate that optical NIR fluorescence imaging can be used to assess neutrophil infiltration into the livers of C57BL/6 mice, a pilot study was initially performed to determine the biodistribution and clearance kinetics of ex vivo labelled Wild Type (WT) neutrophils when given to WT recipient mice. Hepatic neutrophil chemotaxis was stimulated by injuring recipient WT mice with carbon tetrachloride (CCl_4_), which when metabolised by the liver is hepatoxic, causing acute liver injury and hepatic inflammation. To minimise the effect of autofluorescence and to enhance signal to noise ratio, cells were labelled with a Near Infrared (NIR) commercially available fluorophore; CellVue NIR815. Whole body imaging was performed using an excitation and emission filter of 745/820 (Ex/Em). In vivo imaging revealed that the fluorescent signal was detected in the upper abdominal area where the liver is anatomically located. The neutrophils maximally infiltrated the liver by 2 h post cell-administration and the signal was stable for up to 24 h (Supplemental Fig. S1A,B). Neutrophils have a relatively short half-life^19^, therefore at the later time points it was possible that the signal may arise, in part, from dead cells or intrinsic immune cells (macrophages) which may have engulfed the fluorescently labelled neutrophils^20^. Therefore, for all of the following experiments imaging was performed at 2 h post cell administration.

To establish whether ex vivo labelled neutrophils accumulate in the liver irrespective of injury a further pilot experiment was performed. Neutrophil migration was compared between mice injected with olive oil (vehicle control) which does not cause liver damage and those given CCl_4_ to induce liver injury and stimulate an innate immune response. As expected, the in vivo IVIS imaging data showed that the fluorescent signal was lower in olive oil controls. The residual signal observed in the oil treated mouse is expected to be from a low number of neutrophils transiently passing through the liver. In contrast, CCl_4_ injured mice display a much larger photon density map which indicated an increased number of neutrophils infiltrating into the injured liver. CCl_4_ causes cell injury which in turn signals initiation of repair mechanisms which includes recruitment of neutrophils to the site of injury^16^. Importantly, ex vivo imaging confirmed that the fluorescence signal was liver specific with no signal being observed in the spleen, kidneys or lung (Supplemental Fig. S1C).

With the above measures in place, it was important to assess if such method can be applied to differentiate two different neutrophil populations based on their chemotactic properties.

**Figure 1:**
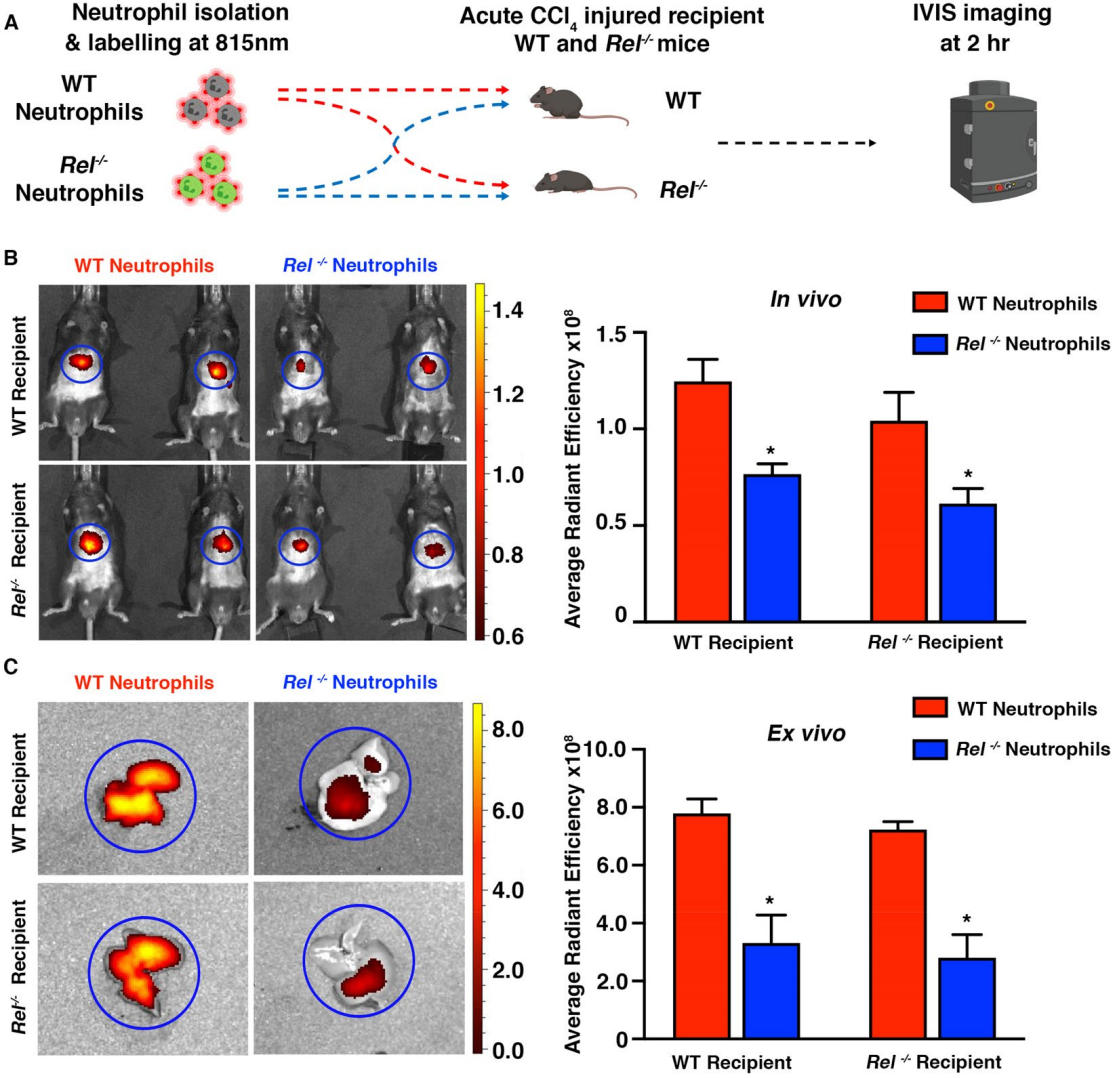
In vivo fluorescent imaging permits monitoring of immune cell recruitment in C57BL/6 mice. (**A**) Schematic diagram showing the experimental design (diagram was created using biorender.com). WT and *Rel*^*−/−*^ neutrophils where isolated from C57BL/6 and *Rel*^*−/−*^ donor mice. All neutrophils were labelled with NIR815 dye. Following labelling, cells were administered intravenously into CCl_4_ injured mice 2 h prior to IVIS imaging. (**B**) Whole body IVIS fluorescence images of 815 nm dye labelled WT or *Rel*^*−/−*^ donor neutrophils tracking to the liver of acute CCl_4_ injured WT or *Rel*^*−/−*^ recipient mice. Graph showing Average Radiant Efficiency of IVIS imaged mice. (**C**) Ex vivo IVIS representative images of livers from recipient mice and graph showing average radiant efficiency of ex vivo imaged livers. In vivo and ex vivo signal was quantified using regions of interest, depicted by blue circles. Images were acquired using 745/820 nm Ex/Em filters. Data are mean ± s.e.m, minimum of n = 7 recipients/group. P values calculated using an unpaired t-test, *P < 0.05.

It has previously been reported that in response to liver injury, mice lacking the c-Rel subunit of the transcription factor nuclear factor‐kappaB (*Rel*^*−/−*^) exhibit an impaired recruitment of neutrophils to the injured liver compared to WT mice^15^. To assess the ability of WT and *Rel*^*−/−*^ neutrophils to migrate to the injured liver, WT and *Rel*^*−/−*^ recipient mice were injured with CCl_4_. Twenty-four hours later, neutrophils were isolated from the bone marrow of naive WT and *Rel*^*−/−*^ donor mice, ex vivo labelled with CellVue NIR815 and then intravenously administered into CCl_4_ injured WT and *Rel*^*−/−*^ recipient mice*.* In vivo fluorescence imaging was performed 2 h post cell administration (Fig. 1A). Prior to administration of the cells, the labelling efficiency between WT and *Rel*^*−/−*^ neutrophils was assessed with IVIS (Ex/Em filters 745/820 nm). Cell imaging confirmed no differences in fluorescence signal between WT and *Rel*^*−/−*^ neutrophils (Supplemental Fig. S1D).

In vivo whole-body IVIS imaging showed that *Rel*^*−/−*^ neutrophils produced significantly lower fluorescent signal compared to WT neutrophils regardless of the genetic background of the recipient mouse (Fig. 1B). Ex vivo imaging confirmed that the signal was liver specific and that *Rel*^*−/−*^ neutrophils display reduced fluorescent signal regardless of recipient genotype (Fig. 1C).

To confirm that the impaired cell migration was due to an intrinsic defect in *Rel*^*−/−*^ neutrophils rather than due to differences in the level of liver injury between WT and *Rel*^*−/−*^ mice, hepatocellular damage was assessed. Comparable elevations of alanine aminotransferase (ALT) and aspartate aminotransferase (AST) in the serum (liver damage markers) and necrotic area assessed by haematoxylin and eosin (H&E) staining confirmed that the livers of WT and *Rel*^*−/−*^ recipient mice were equally damaged (Supplemental Fig. S1E,F).

### Dual fluorescent imaging (DFI) can be used to monitor two cell populations when administered superficially

Currently, using fluorescence imaging to understand the dynamic interplay of two different cell populations requires that both cells are labelled with the same NIR fluorophore and injected into two different recipient mice^21^. Such adoptive transfer experiments increase animal numbers and are difficult to control due to mouse-to-mouse variability and potential inconsistencies in intravenous administration of cells. A limitation of NIR dyes in the far red, such as NIR815, is that these dyes are not compatible with standard fluorescence imaging technologies such as flow cytometry and microscopy, which in turn renders ex vivo data validation at cellular level extremely difficult.

**Figure 2:**
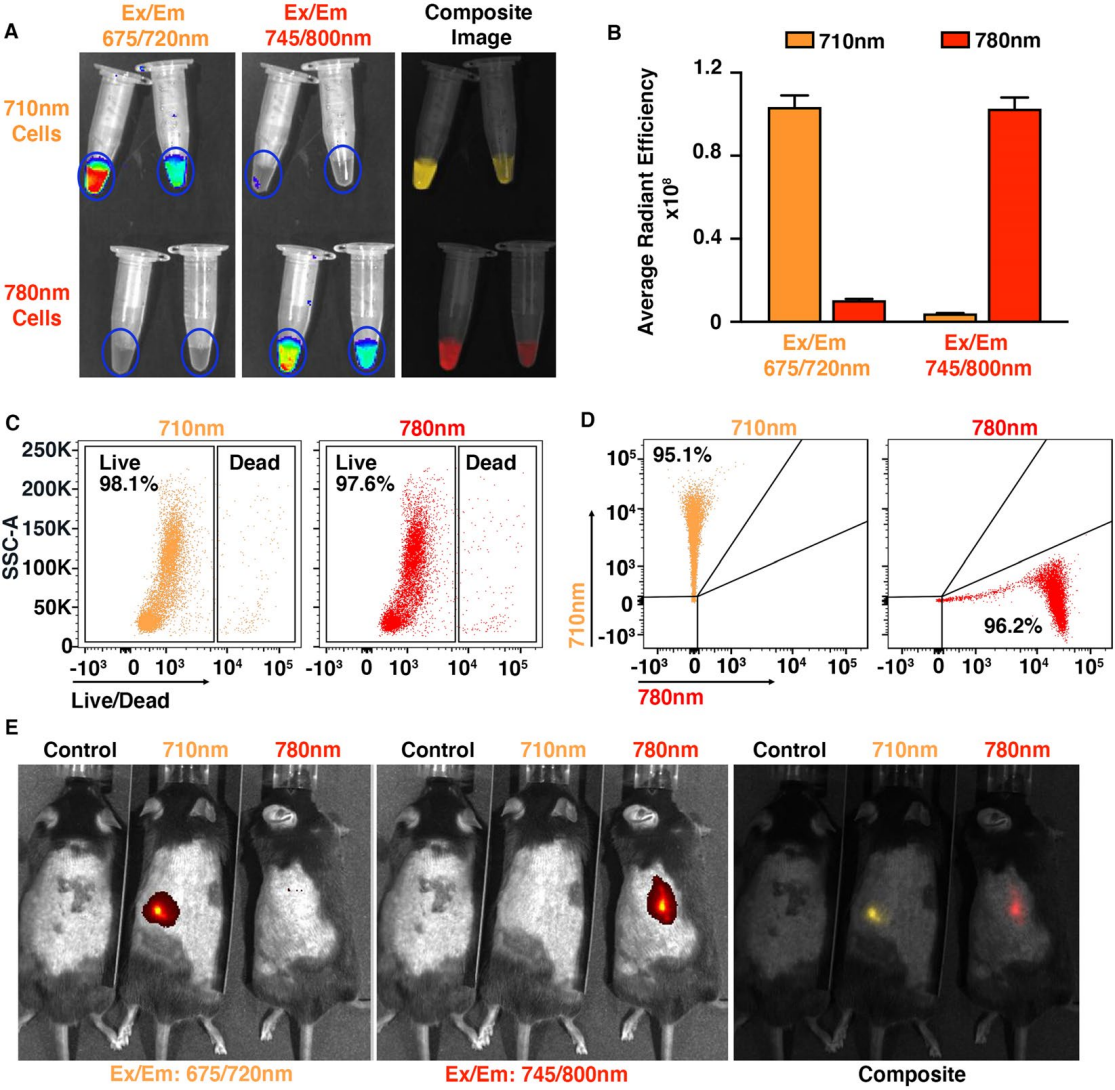
In vitro and superficial in vivo validation of Dual Fluorescent Imaging. (**A**) IVIS images show in vitro spectral un-mixing of neutrophils labelled with either Burgundy (710 nm) or NIR780 (780 nm) using 675/720 and 745/800 nm Ex/Em filters respectively and the corresponding composite image. (**B**) Graph showing average radiant efficiency [p/s/cm^2^/sr]/[µW/cm^2^] of 710 nm and 780 nm fluorophores using 675/720 and 745/800 nm Ex/Em filters. To achieve the same IVIS signal between the two dyes, we used 2 μM of the 710 and 4 μM for the 780 dye. (**C**) Flow cytometry plots showing cell viability and (**D**) labelling efficiency of stained neutrophils. (**E**) In vivo spectral un-mixing of cells labelled with the 710 nm and 780 nm dyes injected into the flank of C57BL/6 mice. Left panel shows IVIS scan acquired at 675/720 nm Ex/Em, middle panel shows IVIS scan at 745/800 nm Ex/Em and the right panel demonstrates in vivo cell separation based on the cell labelling fluorophore. Data are mean ± s.e.m, minimum of n = 3 cell isolations per group.

Because of the above aforementioned challenges, we aimed to develop a methodology which allows two different cell populations to be monitored temporally and spatially within the same animal based on the spectral signature of the cell labelling fluorophore. We identified CellVue NIR780 (780 nm) and CellVue Burgundy (710 nm) as two potential cell labelling candidates which could undergo spectral un-mixing using IVIS. Although the manufacturer of these dyes recommends excitation and emission filters sets, we performed synchronous IVIS imaging for both of these dyes using a broad combination of filters to identify the most appropriate IVIS filters which provide maximum signal intensity and allow for spectral unmixing (Supplemental Fig. S2A). Synchronous IVIS imaging of 780 nm (Ex/Em 745/800) and the 710 nm (Ex/Em 675/720) labelled cells revealed that the two dyes could successfully be spectrally unmixed in vitro*.* (Fig. 2A).

Following this, the concentration of the 710 nm and 780 nm dyes was determined by titrating the dyes to provide concentrations that resulted in a similar IVIS fluorescent signal output for both dyes whilst not affecting cellular viability. Quantification of the fluorescent signal by IVIS imaging revealed that cells labelled with 2 μM of the 710 nm and 4 μM of the 780 nm dye resulted in a similar fluorescence signal output (Fig. 2B). This allowed us to make direct comparisons between two different cell populations when using the above labelling strategy. For flow cytometry neutrophils were identified as both Ly6G and CD11b postivie (Supplemental Fig. S2B). Flow cytometry confirmed that neither fluorophore caused cytotoxicity (Fig. 2C) and that both dyes labelled a similar number of cells (Fig. 2D). To minimise the effect of autofluorescence and depth, dual fluorescent imaging (DFI) was initially tested superficially; 5 million neutrophils labelled with either the 710 nm Burgundy or 780 nm NIR780 dyes were injected subcutaneously into the flank of C57BL/6 WT mice. IVIS imaging confirmed that both dyes could be spectrally unmixed and that the signal generated was comparable (Fig. 2E).

### Dual fluorescent Imaging can be used to monitor cell recruitment to the liver

The biggest challenge with imaging abdominal visceral organs is diet induced autofluorescence^22^. To limit this, mice were fed a chlorophyll free diet for two weeks prior to commencing in vivo imaging (Supplemental Fig. S2C). Potential autofluorescence as a result of stress induced pica^23^ was limited by housing animals in cell sorb bedding, a gypsum treated paper, that produced the least fluorescence signal when IVIS imaged (Supplemental Fig. S2D).

**Figure 3:**
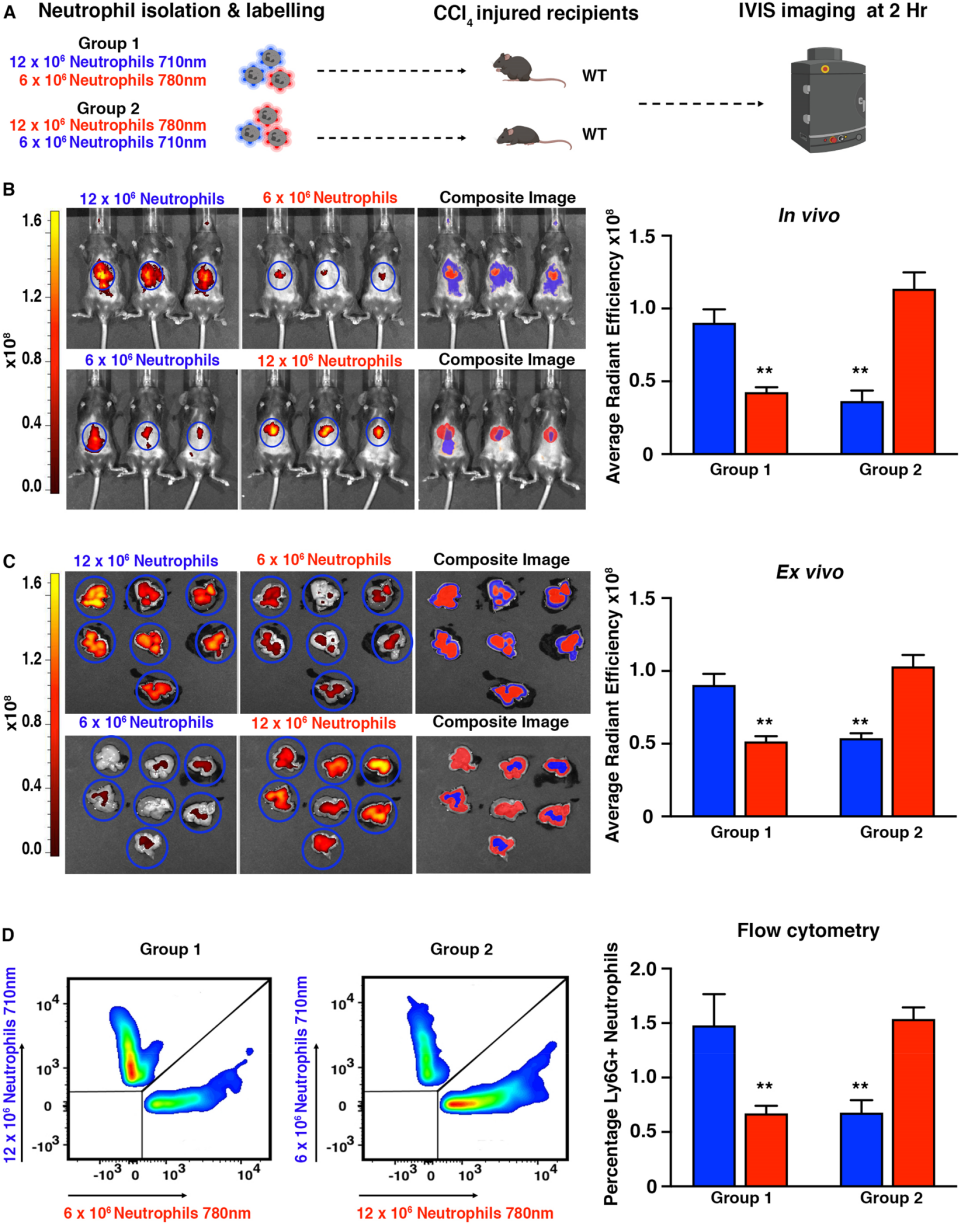
Dual fluorescent imaging in a deep-seated visceral organ. (**A**) Schematic showing the experimental design (diagram was created using biorender.com). (**B**) Whole body IVIS images of 710 nm (left), 780 nm (middle) dye labelled WT donor neutrophils tracking to the liver of acute CCl_4_ injured WT recipient mice. Composite image (right). Graph showing average radiant efficiency [p/s/cm^2^/sr]/[µW/cm^2^] of IVIS imaged mice. (**C**) Ex vivo IVIS images of livers imaged at 710 nm (left), 780 nm (middle) and composite image (right). Graph showing average radiant efficiency from the livers. (**D**) Flow cytometry plots of fluorescently labelled live single cells isolated from CCl_4_ injured livers of WT recipient mice. Graph showing flow cytometry data expressed as a percentage of total immune cells. Data are mean ± s.e.m, minimum of n = 7 recipients per group. P values calculated using two-way ANOVA with Tukey post hoc where **P < 0.01.

To determine whether IVIS in conjunction with CellVue 710 nm and NIR780 could simultaneously monitor two populations of cells in a deep-seated organ, such as the liver, in the same animal we performed the following experiment. Acute CCl_4_ injured mice (Group 1) were injected intravenously with a mixture of fluorescently labelled neutrophils (total of 18 million), of which 12 million were labelled with the 710 nm Burgundy dye and 6 million were labelled with the 780 nm NIR780 dye. To control for differences in fluorescence penetrance, a reciprocal group of mice (Group 2) received a mixture of fluorescently labelled neutrophils (total of 18 million), where 6 million were labelled with the 710 nm burgundy dye and 12 million with the 780 nm NIR780 dye. Mice were then IVIS imaged at 2 h post cell administration (Fig. 3A). Liver enzyme function tests and H&E staining for necrotic area confirmed an equivalent level of injury between both groups of mice (Supplemental Fig. S3A,B).

In vivo imaging showed that cells labelled with both dyes produced a signal in the upper region of the abdominal area where the liver is located. Regardless of the fluorophore used, the IVIS fluorescence signal generated by the fluorophore used to label 12 million neutrophils was generally double that of the fluorescence signal detected when mice were administered 6 million neutrophils (Fig. 3B). Ex vivo imaging confirmed that the signal was liver specific and that the fluorescent signal followed a similar pattern as the in vivo data (Fig. 3C). Composite images show that the dominant fluorescent overlay originates from the fluorophore used to label the higher number of neutrophils (Fig. 3B,C).

Both CellVue Burgundy and CellVue NIR780 were selected due to compatibility with standard flow cytometry filters, which allows rapid isolation of a single cell type from a mixed cell population based on the fluorescence spectra. Most importantly, it provides methodological means of validating in vivo and ex vivo imaging data at a cellular level. The percentage of fluorescently labelled neutrophils isolated from the liver of acute CCl_4_ injured mice was approximately twofold higher in donors receiving 12 million neutrophils, compared to those receiving only 6 million neutrophils, irrespective of the labelling dye used (Fig. 3D), confirming that the fluorescent signal originated from the labelled cells rather than being derived from free dye or cellular debris.

### DFI can be used to monitor biological differences in immune cell recruitment

**Figure 4:**
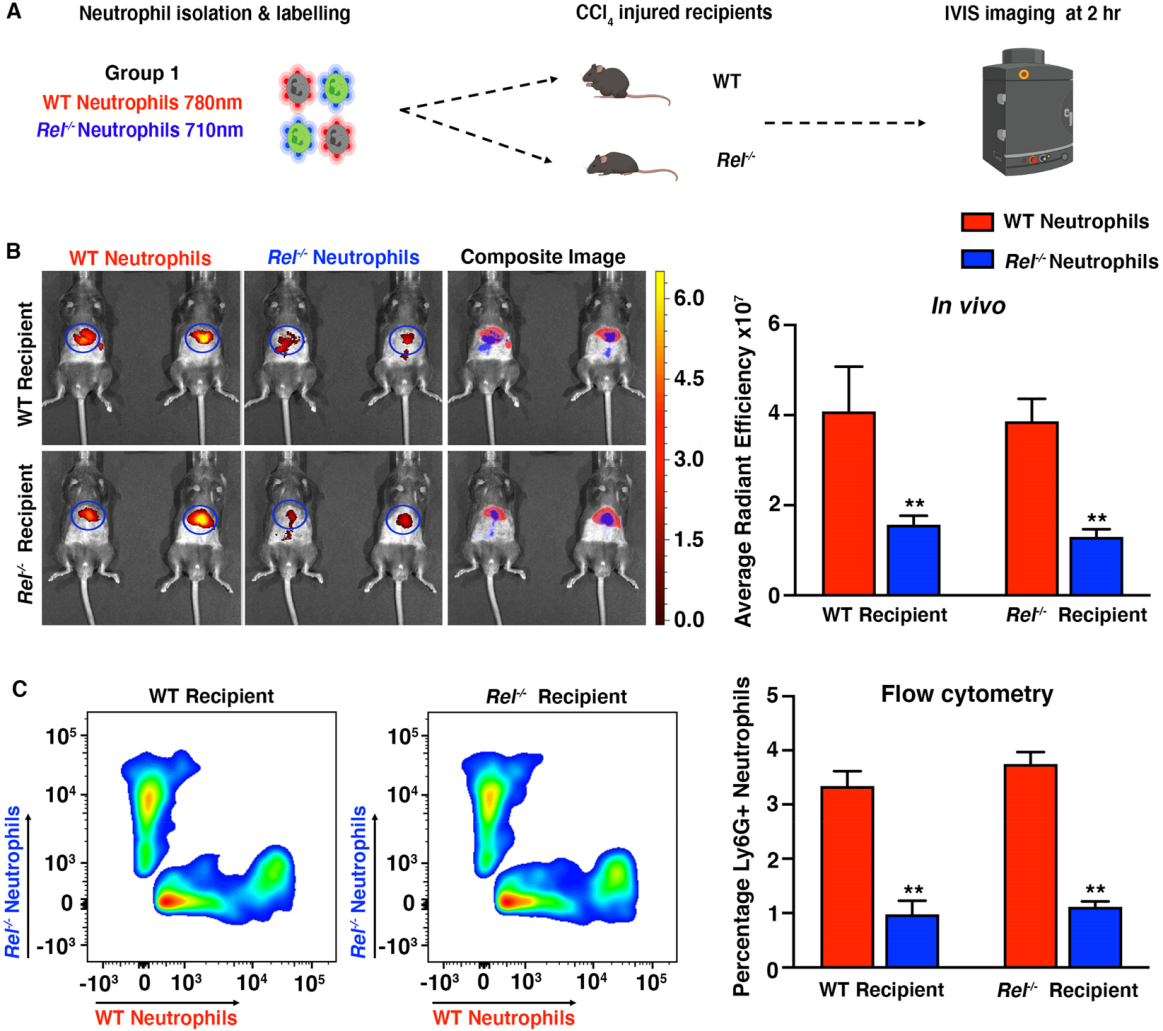
Dual fluorescent imaging can detect biological differences in neutrophil recruitment. (**A**) Schematic showing the experimental design (diagram was created using biorender.com). (**B**) Whole body IVIS images of WT and *Rel*^*−/−*^ recipient mice injected with a mixture of equal numbers of WT and *Rel*^*−/−*^ neutrophils. Left panel shows whole body mouse scans acquired at 745/800 nm Ex/Em. Middle panel shows IVIS images scanned at 675/720 nm Ex/Em and the right panel demonstrates in vivo cell separation based on the cell labelling fluorophore. Graph showing average radiant efficiency [p/s/cm^2^/sr]/[µW/cm^2^] of WT and *Rel*^*−/−*^ recipient mice injected with 780 nm labelled WT neutrophils and 710 nm labelled *Rel*^*−/−*^ neutrophils. (**C**) Flow cytometry plots of fluorescently labelled live single cells isolated from CCl_4_ injured livers of WT recipient *Rel*^*−/−*^ recipient mice injected with 780 nm labelled WT neutrophils and 710 nm labelled *Rel*^*−/−*^ neutrophils. Graph showing flow cytometry data expressed as a percentage of total immune cells. Data are mean ± s.e.m, minimum of n = 4 recipients per group. P values calculated using two-way ANOVA with Tukey post hoc where **P < 0.01.

The in vivo dual fluorescence imaging methodology provided us with the opportunity to assess WT and *Rel*^*−/−*^ neutrophil migration within the same animal. Fluorescently labelled bone marrow derived neutrophils isolated from donor WT and *Rel*^*−/−*^ mice were administered intravenously into CCl_4_ injured WT and *Rel*^*−/−*^ recipient mice (Fig. 4A and Supplemental Fig. S4A). Flow cytometry confirmed similar cell viability and that a similar number of cells were labelled (Supplemental Fig. S4B). As expected, IVIS imaging showed that WT neutrophils migrated in greater numbers to the CCl_4_ injured liver regardless of the genotype of the recipient mice (Fig. 4B and Supplemental Fig. S4C). Flow cytometry confirmed that the number of *Rel*^*−/−*^ neutrophils isolated from CCl_4_ injured livers was significantly lower compared to WT neutrophils (Fig. 4C and Supplemental Fig. S4D). Analysis of H&E stained liver sections and serum transaminase levels indicated that WT and *Rel*^*−/−*^ mice had a comparable level of hepatic injury eliminating the possibility that the difference in cell migration were due to differences in liver damage (Supplemental Fig. S4E,F).

## Discussion

Liver injury is a complex multicellular process that involves crosstalk between different cell types^24^. Chronic inflammation is an underlying cause to the development of chronic liver disease, fibrosis and liver cancer^25^. Therefore, significant efforts have been made to non-invasively monitor hepatic inflammation and test anti-inflammatory agents. In vivo imaging modalities in conjunction with a molecular probe, which is trapped intracellularly, have provided a useful tool for monitoring the dynamics of a single cell population within the same animal^21,26^.

Similar to methodology reported in the literature we have used adoptive transfer experiments, NIR probes and in vivo fluorescence imaging to monitor immune cell recruitment to acute injured liver. The data obtained from a pilot biodistribution experiment showed that the in vivo signal was stable for up to 24 h. However, although IVIS in conjunction with NIR probes provides high signal to noise ratio, this technology lacks resolution to determine cell fate at a low cell number or single cell level. A limitation of NIR probes is that they bind non-covalently to the cell phospholipid bilayer^27,28^. Consequently, it is possible that the signal detected in vivo at the later time points could be generated from either free dye, host immune cells which may have engulfed the free dye or the administered cells.

Because the primary aim of this paper is to establish an in vivo synchronous dual cell tracking method rather than characterise chemotactic properties of neutrophils from different genetic backgrounds, it was decided to image all animals 2 h post cell administration. Nonetheless, this paper provides the methodological foundations to explore cell fate at cellular level using flow cytometry.

Here we report that *Rel*^*−/−*^ neutrophils display an intrinsic defect, which impairs their chemotactic properties to migrate to the CCl_4_ acute injured liver. This is consistent with a previous study which used immunohistochemistry to show that neutrophil recruitment is impaired in CCl_4_ injured *Rel*^*−/−*^ mice^15^.

Adoptive transfer experiments, due to technical limitations, are prone to multiple variables which can have an impact on the final result. Because fluorescently labelled cells are injected into different mice, it is difficult to control for mouse-to-mouse variability, differences in response to injury and operator intravenous cell administration. All of these are major contributory factors in obtaining false positive/negative results. Extra biological variations may be introduced into the dataset and may result in the experimental design being underpowered, consequently resulting in an increase in group numbers, which, in return will lead to more animals being used.

To overcome these challenges, we have optimised an in vivo dual fluorescent imaging method. There are literature reports which demonstrate in vivo multiplexing, the experiments have been performed in BALB/c or nude mice to avoid fine black fur and skin pigmentation^12,29,30^. Melanin skin pigmentation significantly attenuates signal detection^18^, hypothesised to be due to the high absorption coefficient associated with melanin^31^. Therefore, using albino or BALB/c mice restricts the application of these methods to only certain animal models and mouse strains. This can be a major limitation especially when considering the majority of transgenic mice are generated on a C57BL/6 background.

The method described here utilised C57BL/6 mice to image cell migration in a visceral organ such as the liver. Signal attenuation by black fur was minimised by shaving mice. To limit autofluorescence mice were fed chlorophyll free diet. Cell-sorb bedding was used in order to minimise autofluorescence as a result of stress induced pica. Compared to wood shaving and paper, cell-sorb produced the least fluorescent signal. With these measures in place we were able to demonstrate that DFI can be applied to differentiate between WT and *Rel*^*−/−*^ neutrophils based on the cell labelling fluorophore. By using fluorophores which are compatible with flow cytometry, we were able to validate at cellular level that the in vivo fluorescent signal correlates with the number of fluorescently labelled neutrophils.

Dual cell tracking methodology has the potential to be applied to a wide range of biological applications. The methodology could be used in various orthotopic mouse models of disease.

In cell-based therapy DFI would allow assessment of the kinetics and therapeutic capabilities of various cell types within the same animal. Spectral un-mixing would provide a useful tool in drug design and development to both academia and industry to determine the pharmacokinetics of two drug candidates at the same time. DFI can also be used to assess the binding affinity and selectivity of molecular probes to a biological target in vivo.

Technological advances in optical imaging technology (hardware and software) and establishment of in vivo multiplexing protocols permit modelling of complex biological systems. The ability to co-register bioluminescence images with fluorescence scans allows monitoring of various immune cells for their ability to infiltrate orthotopically implanted bioluminescence tumours. Co-registration of fluorescence images with anatomical imaging such as Computerised Tomography allows anatomical localisation of fluorescently labelled cells, peptides and antibodies to a specific organ^32,33^. The diverse biological applications of DFI either alone or in combination with bioluminescence or other imaging modalities, will not only enable researchers to address more complex biological questions using minimally-invasive whole body imaging and test new therapeutic agents, but will ultimately reduce animal use in line with the 3R’s; reduction, replacement and refinement.

To our knowledge this is the first study to report the quantitative characterisation of two different cell populations in the liver of C57/BL6 mice based on the spectral signature of the cell labelling fluorophore. This spectral unmixing methodology has the potential to synchronously monitor the interplay and kinetics of two different cells types in the same animal, as well as monitor therapy response, in many acute or chronic diseases such as tissue inflammation, fibrosis and cancer or neurodegenerative pathologies.

### Ethical approval and study guidelines

All animal experiments were approved by the Newcastle University Ethical Review Committee. All experiments were performed according to the guidelines and regulations described under a UK Home Office licence.

## Supplementary Information

Supplementary Information

## Abbreviations

DFI: Dual fluorescence imaging
ALT: Alanine aminotransferase
AST: Aspartate aminotransferase
CCl_4_: Carbon tetrachloride
FBS: Fetal bovine serum
FFPE: Formalin-fixed, paraffin-embedded
H&E: Haematoxylin and eosin
IVIS: In vivo imaging system
MRI: Magnetic resonance imaging
NIR: Near infrared region
PBS: Phosphate buffered saline
PET: Positron-emission tomography
ROI: Regions of interest
SPECT: Single-photon emission tomography
SNR: Signal to noise ratio
TAF: Tissue autofluorescence
WT: Wild type
