# Supplementary material for: Non-invasive synchronous monitoring of neutrophil migration using whole body near-infrared fluorescence-based imaging
Source: Sci Rep. 2021 Jan 14;11:1415. doi: 10.1038/s41598-021-81097-8 (PMC7809207; doi:10.1038/s41598-021-81097-8)
Supplement: Supplementary file 1 — Supplementary Information [file 41598_2021_81097_MOESM1_ESM.docx]

**Non-invasive synchronous monitoring of neutrophil migration using whole body near-infrared fluorescence-based imaging.**

**Jack Leslie^1^, Stuart M Robinson^1,2^, Fiona Oakley^1^ and Saimir Luli*^1,3^**

*^1^Newcastle Fibrosis Research Group, Biosciences Institute, Faculty of Medical Sciences, Newcastle University, Newcastle upon Tyne, UK.*

*^2^Department of Hepatobiliary Surgery, Newcastle upon Tyne Hospitals NHS Foundation Trust, Newcastle upon Tyne, UK.*

*^3^Preclinical In Vivo Imaging, Biosciences Institute, Faculty of Medical Sciences, Newcastle University, Newcastle upon Tyne, UK.*

**Contact information:** *Corresponding author: Saimir Luli, *Preclinical In Vivo Imaging, Biosciences Institute, Faculty of Medical Sciences,* 4th Floor, William Leech Building, Newcastle University, Framlington Place, Newcastle upon Tyne, NE2 4HH, UK. Tel +44 191 208 5126. Fax +44 191 208 0723. E-mail saimir.luli@newcastle.ac.uk

**Supplementary data**


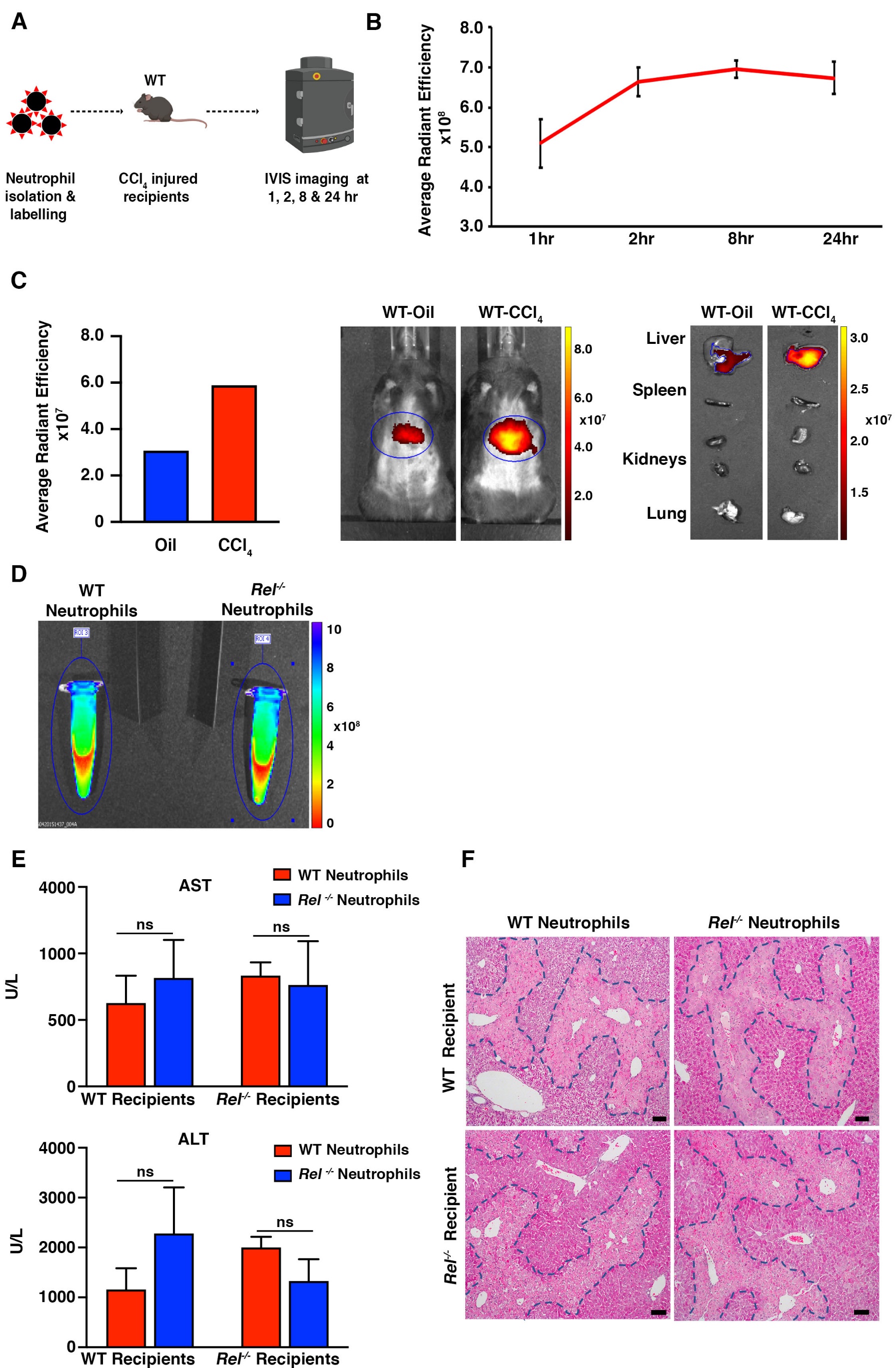


**Supplementary Figure 1: Cell infiltration is liver specific**

(A) Schematic diagram of the experimental design (diagram created using biorender.com). (B) Graph demonstrating cell longitudinal monitoring and signal stability of CellVue NIR815 labelled WT neutrophils. (C) Graph showing Average Radiant Efficiency of IVIS imaged mice followed by representative *in vivo* imaging and *ex vivo* IVIS scans of the liver, spleen kidney and lungs from CCl_4_ and oil treated mice injected with WT neutrophils (10 million) labelled with NIR815. (D) IVIS *in vitro* imaging (745/820 nm Ex/Em) of WT and *Rel^-/-^* neutrophils showing equal cell labelling*.* (E) Graph showing average serum transaminases AST and ALT expressed as units/litre (U/L) from acute CCl_4_ injured WT or *Rel^-/-^* recipient mice where ‘ns’ indicates that no statistical difference was observed following a two-way ANOVA. (F) Photomicrographs (3x3 fields, 100x magnification, scale bar = 200 μm) of haematoxylin and eosin (H&E) stained liver sections from acute CCl_4_ injured WT or *Rel^-/-^* recipient mice, dotted blue lines denote damaged area.

**
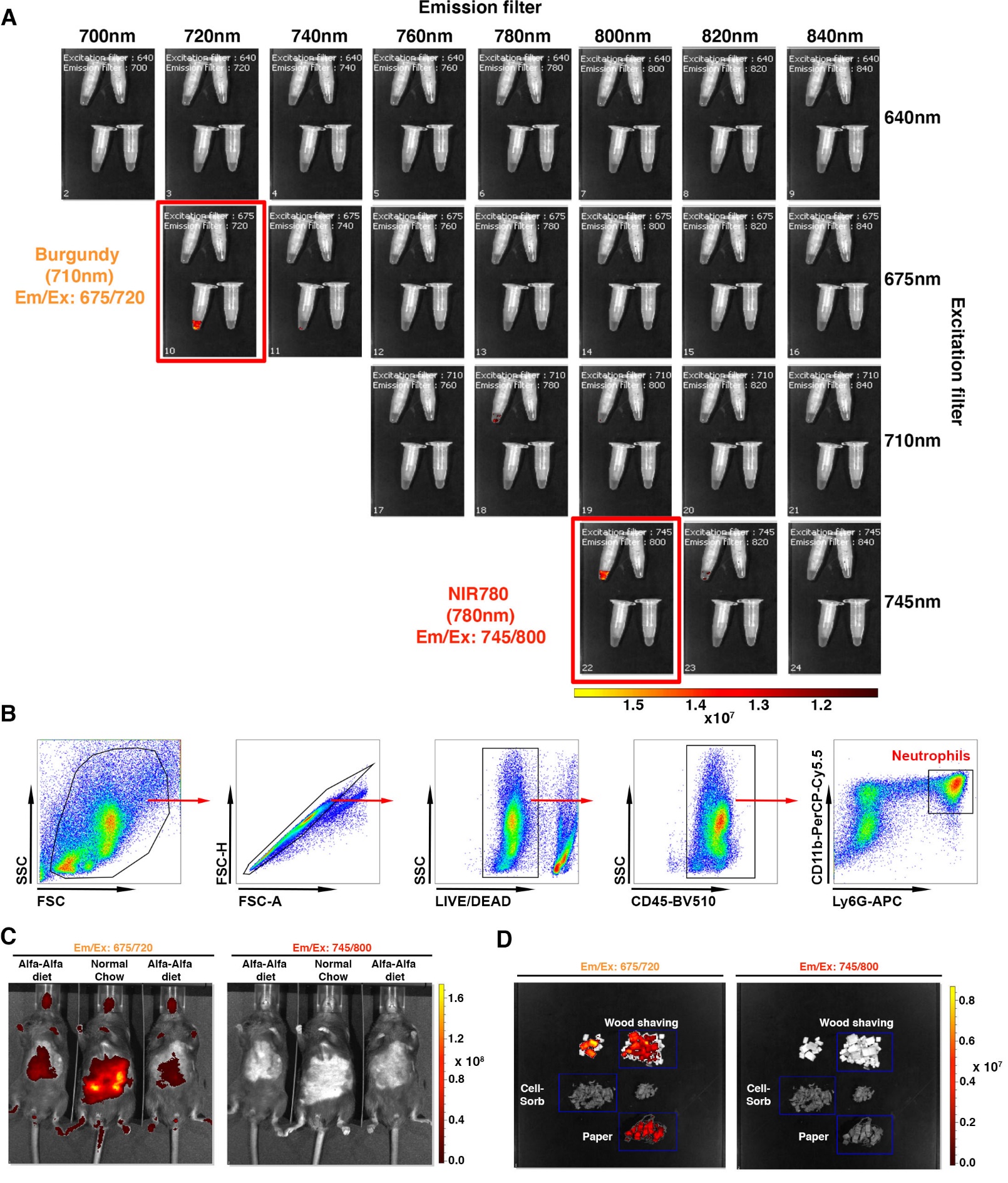
**

**Supplementary Figure 2: Optimisation of dual fluorescence imaging methodology**

(A) IVIS images of cells labelled with either Burgundy or NIR780 scanned synchronously using a combination of excitation and emission filters. For the Burgundy dye an excitation and emission filter of 675/720 nm was found to be the most suitable. Whereas of the NIR780, 745/800 nm excitation and emission filter produced the highest signal intensity. The above channels provided evidence that these 2 dyes can be spectrally separated from each another. (B) Flow cytometry gating strategy; a plot of Forward Scatter-Height (FSC-H) against Side Scatter-Area (FSC-A) was performed to identify single cells. Cells were gated to exclude cellular debris and doublets. Single live immune cells were selected based on positivity for CD45 antibody. Neutrophils were sorted by selecting Ly6G +ve cells.

(C) Whole body IVIS scans of mice fed normal mouse chow or alf-alfa chlorophyll free diet. Mice were imaged at Ex/Em 675/720 and 745/800 nm Ex/Em. (D) IVIS scan of commercially available animal bedding and nesting material. Material were imaged at 675/720 and 745/800 nm Ex/Em.

**
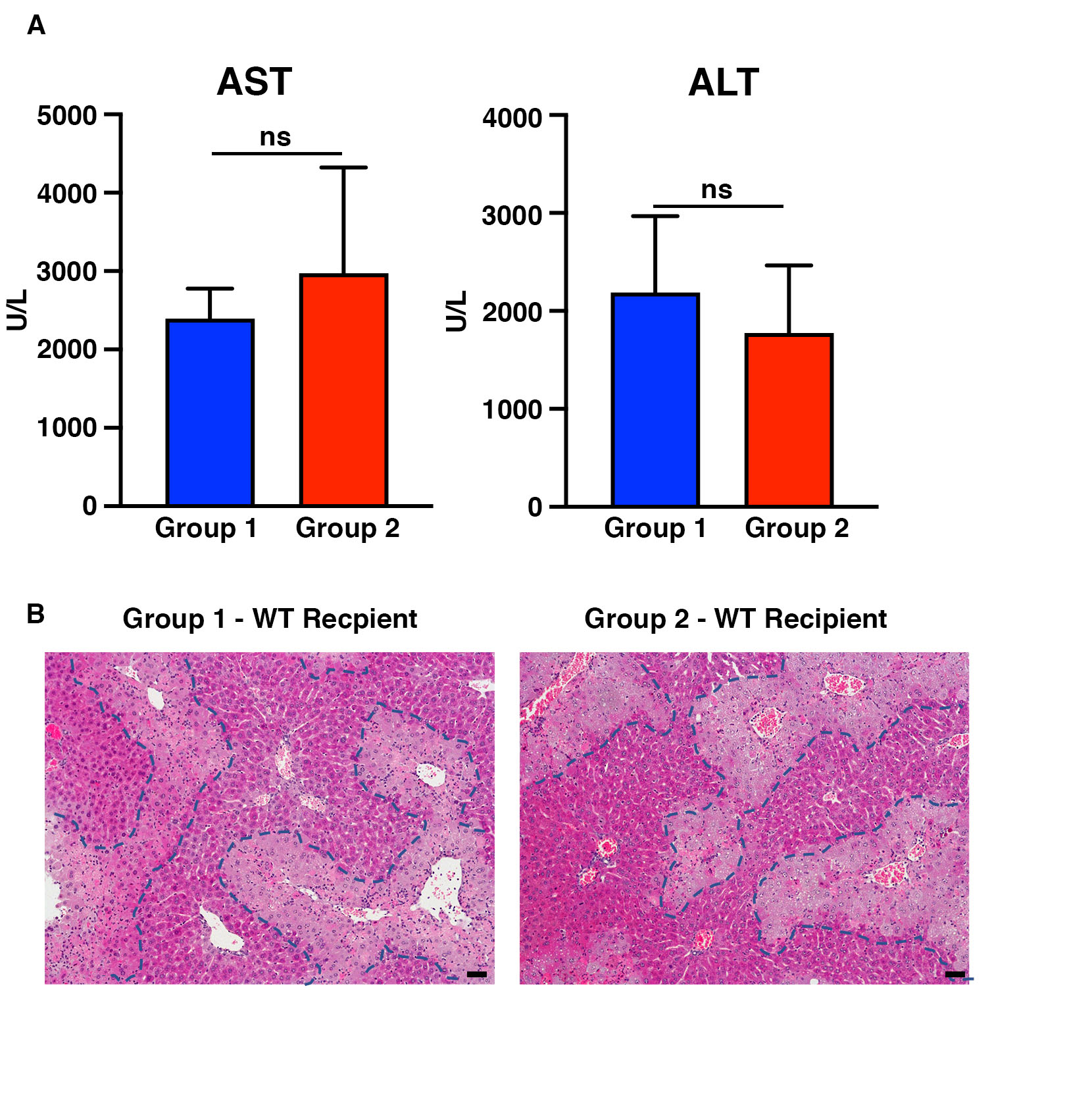
**

**Supplementary Figure 3: Comparable liver damage in WT mice used to validate liver DFI.**

(A) Graph showing average serum transaminases AST and ALT expressed as units/litre (U/L) from acute CCl_4_ injured WT recipient mice. (B) Photomicrographs (3x3 fields, 100x magnification, scale bar = 200 μm) of H&E stained liver sections from acute CCl_4_ injured WT mice, dotted blue lines denote damaged area. Data are means ± s.e.m, minimum n=7 recipients per group, where ‘ns’ indicates that no statistical difference was observed following an unpaired T-test.

**
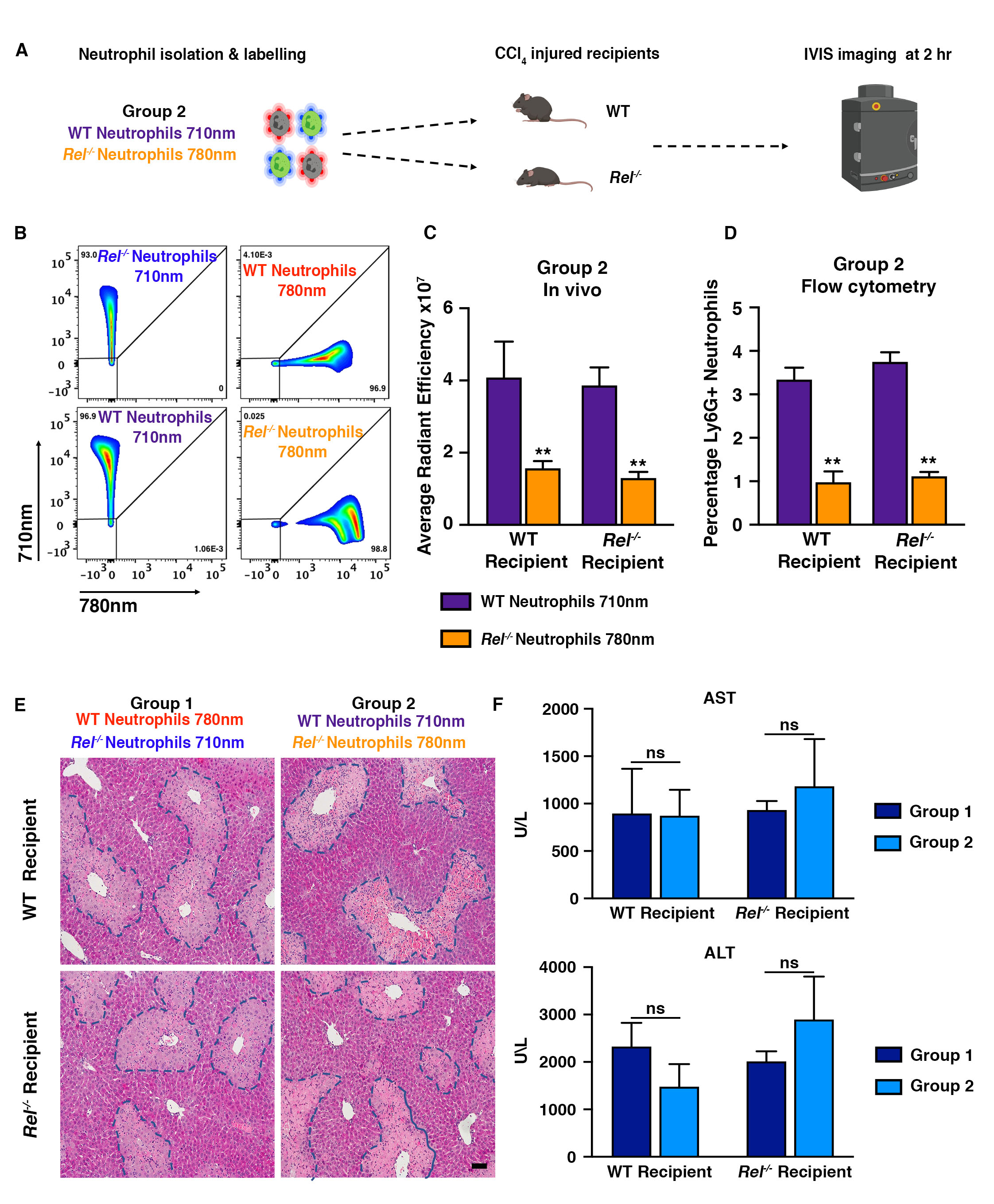
**

**Supplementary Figure 4: WT and *Rel^-/-^* neutrophils have comparable labelling efficiency and WT and *Rel^-/-^* recipients have comparable liver damage**

(A) Schematic overview of the experimental design (diagram created using biorender.com). (B ) Flow cytometry plots showing 710 nm and 780 nm dye labelling efficiency of WT and *Rel^-/-^* neutrophils. (C) Graph showing average radiant efficiency [p/s/cm²/sr]/[µW/cm²] of WT and *Rel^-/-^* recipient mice injected with 710 nm labelled WT neutrophils and 780nm labelled *Rel^-/-^* neutrophils. (D) Graph showing fluorescently labelled live single cells isolated from CCl_4_ injured livers of WT recipient *Rel^-/-^* recipient mice injected with 780 nm labelled WT neutrophils and 710 nm labelled Rel^-/-^ neutrophils as a percentage of total immune cells. (E) Photomicrographs (3x3 fields, 100x magnification, scale bar = 200 μm) of H&E stained liver sections from acute CCl_4_ injured WT mice, dotted blue lines denote damaged area. (F) Graph showing average serum transaminases AST and ALT expressed as units/litre (U/L) from acute CCl_4_ injured WT or *Rel^-/-^* recipient mice. Data are means ± s.e.m, minimum n=4 recipients per group. P values calculated using two-way ANOVA with Tukey post hoc where **P < 0.01 and ‘ns’ indicates that­­­ no statistical difference was observed.
